# A bio-psycho-social approach for frailty amongst Singaporean Chinese community-dwelling older adults – evidence from the Singapore Longitudinal Aging Study

**DOI:** 10.1186/s12877-019-1367-9

**Published:** 2019-12-12

**Authors:** Nigel Teo, Pei Shi Yeo, Qi Gao, Ma Shwe Zin Nyunt, Jie Jing Foo, Shiou Liang Wee, Tze Pin Ng

**Affiliations:** 1 Geriatric Education and Research Institute, Singapore, Singapore; 2 Gerontology Research Programme, Department of Psychological Medicine, National University of Singapore, NUHS Tower Block, 9th Floor, 1E Kent Ridge Road, Singapore, 119228 Singapore; 3 Ministry of Health, Government of Singapore, Singapore, Singapore; 4 Health and Social Sciences Cluster, Singapore Institute of Technology, Singapore, Singapore

**Keywords:** Frailty, Multidimensional frailty, Disability, Nursing home referral, Mortality

## Abstract

**Background:**

Few empirical studies support a bio-psycho-social conceptualization of frailty. In addition to physical frailty (PF), we explored mental (MF) and social (SF) frailty and studied the associations between multidimensional frailty and various adverse health outcomes.

**Methods:**

Cross-sectional and longitudinal analyses were conducted using data from a population-based cohort (SLAS-1) of 2387 community-dwelling Singaporean Chinese older adults. Outcomes examined were functional and severe disability, nursing home referral and mortality. PF was defined by shrinking, weakness, slowness, exhaustion and physical inactivity, 1–2 = pre-frail, 3–5 = frail; MF was defined by ≥1 of cognitive impairment, low mood and poor self-reported health; SF was defined by ≥2 of living alone, no education, no confidant, infrequent social contact or help, infrequent social activities, financial difficulty and living in low-end public housing.

**Results:**

The prevalence of any frailty dimension was 63.0%, dominated by PF (26.2%) and multidimensional frailty (24.2%); 7.0% had all three frailty dimensions. With a few exceptions, frailty dimensions share similar associations with many socio-demographic, lifestyle, health and behavioral factors. Each frailty dimension varied in showing independent associations with functional (Odds Ratios [ORs] = 1.3–1.8) and severe disability prevalence at baseline (ORs = 2.2–7.3), incident functional disability (ORs = 1.1–1.5), nursing home referral (ORs = 1.5–3.4) and mortality (Hazard Ratios = 1.3–1.5) after adjusting for age, gender, medical comorbidity and the two other frailty dimensions. The addition of MF and SF to PF incrementally increased risk estimates by more than 2 folds.

**Conclusions:**

This study highlights the relevance and utility of PF, MF and SF individually and together. Multidimensional frailty can better inform policies and promote the use of targeted multi-domain interventions tailored to older adults’ frailty statuses.

## Background

The multidimensional nature of frailty has been increasingly recognized [1]. However, there remains a dichotomy of frailty definitions in terms of a multidimensional versus a uni-dimensional conceptualization in research and clinical practice. The multidimensional approach to frailty is reflected in instruments such as the (cumulated deficits) Frailty Index [2], which provides a global measure of frailty, and other scales such as the Tilburg Frailty Indicator (TFI) [3, 4] and Edmonton Frail Scale (EFS) [5, 6], which consider physical, psychological and social dimensions of frailty. These multidimensional frailty measures have been evaluated for their construct and predictive validity, have shown good psychometric properties and predicted various adverse health outcomes [3, 7]. Yet, with the exception of the TFI, literature regarding the differential effects of the distinct frailty dimensions within these scales remains obscure [4, 8].

There exist few empirical studies investigating the relationships between each frailty dimension, especially with regards to their independent and relative contributions in predicting functional disability, hospitalization and other adverse health outcomes. Prior studies suggest that physical, mental and social frailty have different associations with known risk factors and predictors [4, 9]. There is little evidence on whether the addition of qualitatively different frailty dimensions to physical frailty can better predict adverse health outcomes. In addition, while the prevalence of these frailty dimensions individually and in combination have been previously described, variations of these estimates in other populations have not been reported partly due to differing frailty operationalization across studies [10]. In line with the World Health Organization’s 2017 global strategy and action plan on ageing and health [11], knowledge regarding a broader conception of frailty beyond physical frailty can aid public health systems in offering tailored policies and interventions to frail older adults. For instance, older adults with physical and mental frailty can benefit from appropriate exercise and cognitive stimulation programs and activities while those with mental and social frailty can benefit from additional cognitive stimulation and appropriate psychosocial support.

In view of this literature gap, we defined three distinct frailty dimensions – physical frailty (PF) [12], mental frailty (MF) [13] and social frailty (SF) [14, 15] – and conducted secondary cross-sectional and longitudinal analyses using data from community-dwelling older adults in the first cohort of the Singapore Longitudinal Aging Studies (SLAS-1). We estimated the individual and combined prevalence of these frailty dimensions, their differential associations with socio-demographic and health-related factors and their independent abilities to predict functional and severe disability, nursing home referral and mortality. We also examined the hypothesis that the addition of MF and SF to PF increases the ability to predict the aforementioned health outcomes.

## Methods

### Participants

Data was obtained from SLAS-1, a population-based longitudinal study investigating aging and health of community-dwelling older Singaporeans aged 55 and above. Individuals with severe physical or mental disabilities were excluded from this study. Detailed methodology is available in a previous publication [16]. The study was approved by the National University of Singapore Institutional Review Board and written informed consent was obtained from all participants.

From a total of 2804 older adults who were recruited at baseline (2003–2005), cross-sectional analyses were conducted using data from 2387 Chinese participants with complete baseline data on designated variables of interest. Longitudinal analyses for functional disability were performed on 1174 participants who could independently complete instrumental or basic activities of daily living (IADL or ADL) at baseline and who had complete follow-up data on IADL and ADL. During the next follow-up (2005–2007), 859 participants dropped out or had missing data for IADL or ADL. Another 354 participants were excluded as they required assistance in one or more IADL or ADL at baseline. Longitudinal analyses for mortality and nursing home referral were conducted for all 2387 participants.

### Baseline frailty measures

***Mental frailty (MF)*** consisted of three components:

1. *Cognitive impairment* as determined using the validated Chinese version of the Mini-Mental State Examination (CMMSE), with scores ranging from 0 to 30 [17]. Higher scores reflect better cognitive functioning. Cognitive impairment was defined as having a CMMSE score equivalent to or lower than 23.
2. *Low mood* was indicated by the presence of any of the following:
  - A score of five or above on the Geriatric Depression Scale, which has been validated for use among Singaporean older adults [18].
  - Answering “None of the time” for the SF-12 question: “Have you felt calm and peaceful for the past four weeks?”
  - Answering “All of the time” for the SF-12 question: “Have you felt downhearted and low for the past four weeks?”
3. *Poor self-rated health* as assessed via the question, “In general, would you say your health is excellent, very good, good, fair, or poor?” Self-rated health was included as a component of MF as it is a rating of one’s overall health status that is driven by cognitive and psychological processes [19].

Scores were assigned to each MF component (1 = present, 0 = absent). Participants with summed scores of one or more were deemed to have MF.

***Social frailty (SF)*** was assessed via socio-demographic variables and self-reported survey questionnaires related to living arrangements, educational attainment, socio-economic status and social network and support. Briefly, the criteria are listed below:

1. Living alone: Yes = 1, No = 0
2. No education: Yes = 1, No = 0
3. Absence of a confidant: Yes = 1, No = 0
4. Infrequent visits or calls with family, friends or loved ones (assessed via two different questions) (none or no more than once a year), or receives little help when required (none to a very little): Having any of the three criterion = 1, else = 0.
5. Infrequent social activities across 6 activity categories: rarely or not at all participate in all categories of social activities = 1, else = 0.
6. Financial difficulty: Limited to a great extent in ability to pay for necessary medical expenses = 1, else = 0.
7. Socio-economic deprivation as indexed by housing type, which has been found to predict readmission risk and increased utilization of hospital services in Singapore [20]: Living in 1–2 room public housing = 1, else = 0

Additional details regarding this operationalization are available in a previous publication [15]. Scores were assigned to each SF indicator (1 = present, 0 = absent). Participants with summed scores of two or more were categorized as having SF. The SF operationalization was shown to predict prevalent IADL and severe disability [15].

***Physical frailty (PF)*** was based on the Fried’s criteria used in the Cardiovascular Health Study, with operational modifications detailed in previous publications and shown to predict IADL-ADL disability, depression, hospitalization and poor quality of life [21, 22]. Scores were assigned to each of the five components (1 = present, 0 = absent). Participants were categorized as frail (score = 3–5), pre-frail (score = 1–2) or robust (score = 0) using the summed score.

### Adverse health outcomes

Ability to perform IADL or ADL was assessed via self-reports [23, 24]. ***Functional disability*** was defined as requiring assistance on one or more IADL or ADL item(s). ***Severe disability*** was denoted by dependency on three or more ADL items, which in Singapore often necessitates formal help in nursing home care placement and qualifies for disability insurance payouts.

***Nursing home referral*** data between 30th August 2010 and 24th February 2018 was obtained via computerized record linkage with the Ministry of Health (MOH)‘s national database. Referral coordinators assessed the older adult’s care needs before making a referral for nursing home placement. Social factors, such as the availability of a caregiver, play a substantial role in prioritizing nursing home referral and placement. Individuals who receive nursing home services via such referrals are assessed for eligibility for government subvention via means testing.

***Mortality*** data (date and cause of mortality) up to the end of November 2016 was obtained via computerized record linkage with the National Death Registry of Singapore through MOH’s National Disease Registry Office.

### Other variables

***Socio-demographic*** data included age and gender. ***Medical morbidity*** was determined through self-reported doctor’s diagnosis and treatment for 16 specified or other medical condition(s) in the past year, medications used, body mass index, blood pressure, spirometry measurements, blood tests for fasting glucose, lipid panels and creatinine for estimated glomerular filtration rate. Participants with three or more medical conditions were deemed to have medical comorbidity. ***Lifestyle variables*** included self-reported current and past history of smoking and daily alcohol drinking. ***Hospitalization and physician visits*** were assessed via self-reported hospitalizations and physician visits for the above medical conditions over the previous year. ***Polypharmacy*** was defined as taking six or more medications and was determined through self-reported medication intake. ***Nutritional risk*** was defined as scoring three or more on the 10-item Nutrition Screening Initiative [25]. ***Hearing impairment*** was assessed via self-reports and the standard whisper test while ***visual impairment*** was defined as having corrected binocular vision worse than 20/40 [26].

### Statistical analyses

Pearson χ^2^ and one-way ANOVAs were used to examine differences in baseline characteristics across the three different frailty dimensions. For each adverse health outcome, odds ratios (ORs) or hazard ratios (HRs) and their 95% confidence intervals were estimated for each frailty dimension and for six different categories of PF (Robust, Pre-frail, Frail) in combination with MF and/or SF: (1) Robust without SF or MF, (2) Robust with MF and/or SF, (3) Pre-frail without MF or SF, (4) Pre-frail with MF and/or SF, (5) Frail without MF or SF, (6) Frail with MF and/or SF. Estimated ORs and HRs were adjusted for age, gender, medical comorbidity and other frailty dimensions, where relevant. Receiver Operating Characteristic (ROC) analyses were used to compare the predictive abilities of PF, MF, SF and combined PF-SF(PSF), PF-MF(PMF) and PF-MF-SF(PMSF) dimensions for the aforementioned adverse health outcomes. A two-sided *p*-value of .05 was considered statistically significant. Analyses were performed using STATA version 14 [27].

## Results

**Fig. 1:**
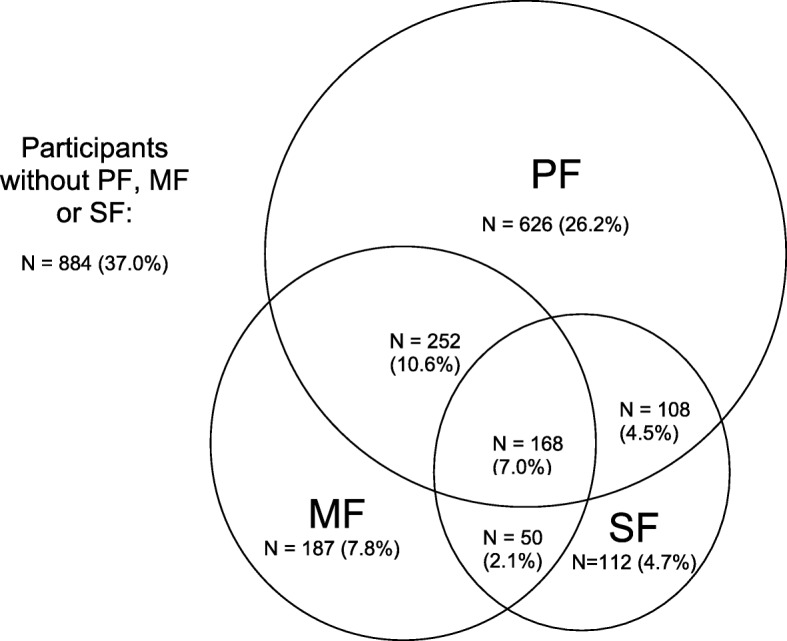
Prevalence and Co-occurrence of Frailty Dimensions in the SLAS-1 Cohort (*N* = 2387). Physical Frailty (PF) is defined as one or more of Fried’s criteria; Mental Frailty (MF) is defined as having one or more MF Criteria; Social Frailty (SF) is defined as having two of more of the SF Criteria

The mean age of the study sample was 66.1 years (±7.61), 63.3% were female and 25.2% were single, divorced or widowed at baseline. The prevalence of each frailty dimension and overlaps across dimensions is shown in Fig. 1. Overall, 37.0% did not have any of the three frailty dimensions while the prevalence of any frailty dimension was 63.0%. PF (i.e., physical pre-frailty and frailty) was present in 48.3% (26.2% were PF alone, 10.6% with MF and 4.5% with SF). The prevalence of MF was 27.5% (7.8% were MF alone, 10.6% with PF and 2.1% with SF). SF was present in 18.3% (4.7% were SF alone. 4.5% with PF, 2.1% with MF). A total of 7.0% had all three frailty dimensions.

### Inter-relationships between frailty dimensions

There were clear associations among the three frailty dimensions (Table 1). PF was strongly associated with psychological and social functioning components of MF and SF while MF was strongly associated with physical and social functioning components of PF and SF. SF was strongly associated with physical and mental functioning components in PF and MF.

**Table 1:** Prevalence of components for social, mental and physical frailty in SLAS-1 Chinese older adults

Variables	Total	Physical Frailty (PF)			Mental Frailty (MF)			Social Frailty (SF)		
	*N* = 2387 (%)	No PF (*N* = 1233)	PF (*N* = 1154)	*p*	No MF (*N* = 1730)	MF (*N* = 657)	*p*	No SF (*N* = 1949)	SF (*N* = 438)	*p*
Fried Shrinking	209 (8.8)	0 (0.0)	209 (18.1)	< 0.001	127 (7.3)	82 (12.5)	< 0.001	146 (7.5)	63 (14.4)	< 0.001
Fried Low Physical Activity	635 (26.6)	0 (0.0)	635 (55.0)	< 0.001	439 (25.4)	196 (29.8)	0.028	495 (25.4)	140 (32.0)	0.005
Fried Weak	471 (19.7)	0 (0.0)	471 (40.8)	< 0.001	256 (14.8)	215 (32.7)	< 0.001	316 (16.2)	155 (35.4)	< 0.001
Fried Slow	77 (3.2)	0 (0.0)	77 (6.7)	< 0.001	29 (1.7)	48 (7.3)	< 0.001	42 (2.2)	35 (8.0)	< 0.001
Fried Exhaust	206 (8.6)	0 (0.0)	206 (17.9)	< 0.001	73 (4.2)	133 (20.2)	< 0.001	147 (7.5)	59 (13.5)	< 0.001
Cognitive Impairment	282 (11.8)	77 (6.2)	205 (17.8)	< 0.001	0 (0.0)	282 (42.9)	< 0.001	141 (7.2)	141 (32.2)	< 0.001
Low Mood	430 (18.0)	164 (13.3)	266 (23.1)	< 0.001	0 (0.0)	430 (65.5)	< 0.001	314 (16.1)	116 (26.5)	< 0.001
Poor Health Status	59 (2.5)	15 (1.2)	44 (3.8)	< 0.001	0 (0.0)	59 (9.0)	< 0.001	38 (2.0)	21 (4.8)	0.001
Live Alone	174 (7.3)	79 (6.4)	95 (8.2)	0.087	114 (6.6)	60 (9.1)	0.033	61 (3.1)	113 (25.8)	< 0.001
No Education	453 (19.0)	169 (13.7)	284 (24.6)	< 0.001	211 (12.2)	242 (36.8)	< 0.001	215 (11.0)	238 (54.3)	< 0.001
Infrequent Contact	487 (20.4)	215 (17.4)	272 (23.6)	< 0.001	296 (17.1)	191 (29.1)	< 0.001	246 (12.6)	241 (55.0)	< 0.001
Infrequent Social Activities	326 (13.7)	158 (12.8)	168 (14.6)	0.215	226 (13.1)	100 (15.2)	0.170	174 (8.9)	152 (34.7)	< 0.001
Financial Difficulty	234 (9.8)	110 (8.9)	124 (10.8)	0.134	127 (7.3)	107 (16.3)	< 0.001	98 (5.0)	136 (31.1)	< 0.001
Absence of Confidant	117 (4.9)	34 (2.8)	83 (7.2)	< 0.001	58 (3.4)	59 (9.0)	< 0.001	27 (1.4)	90 (20.6)	< 0.001
Socio-economic Deprivation	150 (6.3)	35 (2.8)	115 (10.0)	< 0.001	62 (3.6)	88 (13.4)	< 0.001	31 (1.6)	119 (27.2)	< 0.001

Footnotes: Independent sample t-tests were conducted for continuous variables, chi-square for categorical variables

### Socio-demographic, lifestyle, and health and behavioral profiles

Participants with PF, MF and/or SF were more likely to be older, female, single, divorced or widowed, more likely to be smokers, but less likely to be drinkers, more likely to be at risk of malnutrition, to be underweight and to have low cholesterol; to have stroke, asthma/COPD, chronic kidney disease, neurological and psychiatric disorders and visual impairment (Table 2). Prevalence of obesity, high blood pressure, coronary heart disease, cardiac disease and metabolic syndrome was significantly higher in participants with PF as compared to those without PF. The prevalence of these variables, however, were similar regardless of one’s MF or SF status. Having MF was uniquely associated with significantly higher prevalence of arthritis and hospitalizations in the past year. As compared to those without PF or MF, participants with PF or MF reported significantly higher prevalence of diabetes, medical comorbidity, polypharmacy and physician visits. Of note, these variables did not differ by SF status.

**Table 2:** Baseline characteristics of SLAS-1 Chinese older adults by physical, mental and social frailty status

Variables	Total	Physical Frailty (PF)			Mental Frailty (MF)			Social Frailty (SF)		
	*N* = 2387 (%)	No PF (*N* = 1233)	PF (*N* = 1154)	*p*	No MF (*N* = 1730)	MF (*N* = 657)	*p*	No SF (*N* = 1949)	SF (*N* = 438)	*p*
Age, mean (SD) years	66.1 (7.61)	64.2 (6.36)	68.0 (8.34)	< 0.001	65.1 (6.99)	68.5 (8.61)	< 0.001	65.0 (7.31)	69.3 (8.12)	< 0.001
Male	876 (36.7)	493 (40.0)	383 (33.2)	0.001	668 (38.6)	208 (31.7)	0.002	748 (38.12)	133 (30.4)	0.002
Single, Divorced Or Widowed	603 (25.2)	237 (19.2)	365 (31.6)	< 0.001	378 (21.9)	224 (34.1)	< 0.001	385 (19.8)	217 (49.5)	< 0.001
Current smoking (versus others)	154 (6.5)	61 (5.0)	93 (8.1)	0.002	101 (5.8)	53 (8.1)	0.047	113 (5.8)	41 (9.4)	0.006
Current and past smoking	401 (16.8)	191 (15.5)	210 (18.2)	0.080	267 (15.4)	134 (20.4)	0.004	296 (15.2)	105 (24.0)	< 0.001
Alcohol drinking	258 (10.8)	168 (13.6)	90 (7.8)	< 0.001	210 (12.1)	48 (7.3)	0.001	223 (11.4)	25 (8.0)	0.036
NSI Risk (3 and above)	1185 (49.6)	527 (42.7)	658 (57.0)	< 0.001	729 (42.1)	456 (69.4)	< 0.001	911 (46.7)	274 (62.6)	< 0.001
Obese (BMI ≥ 27.5)	310 (13.0)	134 (10.9)	176 (15.3)	0.001	222 (12.8)	88 (13.4)	0.696	252 (12.9)	58 (13.3)	0.834
Underweight (BMI ≤ 18.5)	133 (5.6)	0 (0.0)	133 (11.5)	< 0.001	81 (4.7)	52 (7.9)	0.002	99 (4.8)	40 (9.1)	< 0.001
Hypertension	1340 (56.1)	656 (53.2)	684 (59.3)	0.003	953 (55.1)	387 (58.9)	0.093	1073 (55.1)	267 (61.0)	0.024
High Blood Pressure	1087 (45.5)	532 (43.2)	555 (48.1)	0.015	770 (44.5)	317 (48.3)	0.101	886 (45.5)	201 (45.9)	0.87
High Cholesterol	1017 (42.9)	512 (42.0)	505 (43.9)	0.339	733 (42.7)	284 (43.5)	0.725	852 (44.1)	165 (37.7)	0.014
Low cholesterol	179 (7.5)	78 (6.3)	101 (8.8)	0.025	118 (6.8)	61 (9.3)	0.041	135 (6.93)	44 (10.1)	0.025
Diabetes	411 (17.2)	180 (14.6)	231 (20.0)	< 0.001	276 (16.0)	135 (20.6)	0.008	329 (16.9)	82 (18.7)	0.356
Coronary heart disease	123 (5.2)	48 (3.9)	75 (6.5)	0.004	86 (5.0)	37 (5.6)	0.514	94 (4.8)	29 (6.6)	0.124
Cardiac Disease	194 (8.1)	81 (6.6)	113 (9.8)	0.004	130 (7.5)	64 (9.7)	0.075	151 (7.8)	43 (9.8)	0.152
Stroke	87 (3.6)	23 (1.9)	64 (5.6)	< 0.001	45 (2.6)	42 (6.4)	< 0.001	62 (3.2)	25 (5.7)	0.011
Cancer	43 (1.8)	23 (1.9)	20 (1.7)	0.808	31 (1.8)	12 (1.8)	0.955	35 (1.8)	8 (1.8)	0.965
Arthritis	325 (13.6)	151 (12.3)	173 (15.0)	0.052	217 (12.6)	107 (16.3)	0.016	263 (13.5)	61 (13.9)	0.823
Asthma/COPD	596 (25.0)	270 (21.9)	326 (28.3)	< 0.001	381 (22.0)	215 (32.7)	< 0.001	460 (23.6)	136 (31.1)	0.001
Hip Fracture	20 (0.8)	5 (0.4)	15 (1.3)	0.017	8 (0.5)	12 (1.8)	0.001	13 (0.7)	7 (1.6)	0.053
Chronic kidney disease	993 (42.0)	428 (34.9)	565 (49.7)	< 0.001	661 (38.5)	332 (51.5)	< 0.001	777 (40.2)	216 (50.1)	< 0.001
Metabolic Syndrome	656 (27.7)	316 (25.8)	340 (29.8)	0.032	464 (27.1)	192 (29.5)	0.220	526 (27.2)	130 (30.0)	0.246
Neuropsychiatric illness (*N* = 2384)	87 (3.7)	33 (2.7)	54 (4.7)	0.009	39 (2.3)	48 (7.3)	< 0.001	60 (3.1)	27 (6.2)	0.002
Hearing Impairment	64 (2.7)	27 (2.2)	37 (3.2)	0.124	32 (1.9)	32 (4.9)	< 0.001	40 (2.1)	24 (5.5)	< 0.001
Visual Impairment	809 (33.9)	332 (26.9)	477 (41.3)	< 0.001	548 (31.7)	261 (39.7)	< 0.001	642 (32.9)	167 (38.1)	0.038
Comorbidity	1018 (42.65)	451 (36.58)	567 (49.13)	< 0.001	702 (40.58)	316 (48.10)	0.001	820 (42.07)	198 (45.21)	0.231
Hospitalization for any condition in the past year	95 (4.0)	40 (3.2)	55 (4.8)	0.057	57 (3.3)	38 (5.8)	0.005	81 (4.2)	14 (3.2)	0.353
Polypharmacy (taking 6 or more drugs)	254 (10.6)	99 (8.0)	153 (13.3)	< 0.001	155 (9.0)	97 (14.8)	< 0.001	207 (10.6)	45 (10.3)	0.831
Physician Visit in the past year (SD)	4.07 (5.40)	3.66 (4.51)	4.50 (6.18)	< 0.001	3.83 (4.56)	4.71 (7.12)	< 0.001	4.04 (4.75)	4.21 (7.65)	0.28

Footnotes: *Metabolic syndrome* was defined using the International Diabetes Federation (IDF) criteria^25^: central obesity (waist circumference ≥ 90 for male and ≥ 80 for female) plus at least 2 CVRF: raised triglyceride level (≥150 mg/dL (1.7 mmol/L) or specific treatment for this lipid abnormality); reduced high density lipoprotein cholesterol (< 40 mg/dL (1.03 mmol/L) in males and < 50 mg/dL (1.29 mmol/L) in females or specific treatment for this lipid abnormality); raised blood pressure (BP) (systolic BP ≥ 130 or diastolic BP ≥ 85 mmHg, or treatment of previously diagnosed hypertension), and raised fasting plasma glucose (≥ 100 mg/dL or 5.6 mmol/L), or previously diagnosed type 2 diabetes)

Chronic kidney disease: eGFR (< 60 mL/min/1.73 m^2;^ COPD: FEV1/FVC < 0.70

Neuropsychiatric illnesses: depression, psychosis, anxiety, dementia, Parkinson’s disease

Significance Tests: independent sample t-tests were conducted for continuous variables, chi-square for categorical variables

### Frailty dimensions and disability, nursing home referral and mortality

In cross-sectional analyses (Table 3), adjusted for age, gender, medical comorbidity and other frailty dimensions, prevalent functional disability was significantly predicted by MF (OR = 1.78, *p* < .001) and PF (OR = 1.73, *p* < .001), but not SF. Prevalent severe disability was predicted by all three frailty dimensions after controlling for age, gender, comorbidity and other frailty dimensions (ORs = 2.19–7.34, *p*s = < .001–.048). In longitudinal analyses (Table 4), incident functional disability was significantly higher for PF (OR = 1.49, *p* = .044), but not SF and MF. Only SF significantly predicted nursing home referral after adjusting for age, gender, comorbidity and the two other frailty dimensions (OR = 3.43, *p* < .001). All frailty dimensions significantly predicted mortality after adjustment, with the highest and lowest HRs found for PF (HR = 1.51, *p* < .001) and SF respectively (HR = 1.32, *p* = .018).

**Table 3:** Cross-sectional analyses of baseline MF, SF and PF with prevalent functional and severe disability

	Functional Disability						Severe Disability					
	N	%	OR (95% CI) Un-adjusted	P	OR (95% CI) Adjusted^1^	P	N	%	OR (95% CI) Un-adjusted	P	OR (95% CI) Adjusted^1^	P
Total	2387						2387					
Physical frailty	382/1154	33.1	2.45 (2.02–2.98)	< 0.001	1.73 (1.40–2.25)	< 0.001	28/1154	2.4	15.31 (3.64–64.39)	< 0.001	7.34 (1.68–32.04)	0.008
Mental frailty	244/657	37.1	2.37 (1.95–2.89)	< 0.001	1.78 (1.43–2.23)	< 0.001	24/657	3.7	10.89 (4.43–26.77)	< 0.001	5.52 (2.15–14.22)	< 0.001
Social frailty	155/438	35.4	1.91 (1.53–2.39)	< 0.001	1.26 (0.97–1.63)	0.078	16/438	3.7	5.24 (2.54–10.82)	< 0.001	2.19 (1.00–4.76)	0.048
	2387						2387					
Robust without MF or SF	144/884	16.3	1.00		1.00		2/884	0.2	1.00		1.00	
Robust with MF and/or SF	63/349	18.1	1.13 (0.82–1.57)	0.456	1.16 (0.82–1.63)	0.404	0/349	0.0	NE	–	NE	–
Pre-frail without MF or SF	136/611	22.3	1.47 (1.13–1.91)	0.004	1.31 (1.00–1.74)	0.053	2/611	0.3	1.45 (0.20–10.31)	0.711	1.39 (0.19–9.98)	0.741
Pre-frail with MF and/or SF	183/462	39.6	3.37 (2.60–4.36)	< 0.001	2.58 (1.94–3.43)	< 0.001	8/462	1.7	7.77 (1.64–36.75)	0.010	6.99 (1.43–34.01)	0.016
Frail without SF or MF	8/15	53.3	5.87 (2.10–16.45)	0.001	2.88 (0.99–8.40)	0.052	1/15	6.7	31.50 (2.70–367.90)	0.006	25.72 (2.10–314.94)	0.011
Frail with MF and/or SF	55/66	83.3	25.69 (13.13–50.29)	< 0.001	15.78 (7.76–32.09)	< 0.001	17/66	25.8	153 (34.37–681.01)	< 0.001	125.17 (25.97–603.17)	< 0.001
Robust without MF or SF	144/884	16.3	1.00		1.00		2/884	0.2	1.00		1.00	
Robust with MF and/or SF	63/349	18.1	1.13 (0.82–1.57)	0.456	1.15 (0.82–1.62)	0.412	0/349	0.0	NE	–	NE	–
Pre-frail or Frail without SF or MF	144/626	23.0	1.54 (1.19–1.99)	0.001	1.34 (1.02–1.77)	0.036	3/626	0.5	2.12 (0.35–12.75)	0.410	1.82 (0.30–11.06)	0.514
Pre-frail or Frail with MF and/or SF	238/528	45.1	4.22 (3.29–5.40)	< 0.001	3.10 (2.36–4.08)	< 0.001	25/528	4.7	21.92 (5.17–92.92)	< 0.001	15.37 (3.47–67.98)	< 0.001

^1^Models for Physical frailty, mental frailty and social frailty include: gender, age, comorbidity, physical frailty, mental frailty and social frailty. Models for all other predictors include: gender, age and comorbidity

**Table 4:** Longitudinal analyses of MF, SF and PF with functional disability, nursing home referral and mortality

	Functional Disability				Nursing Home Referral				Mortality			
	N	%	OR (95% CI)	P	N	%	OR (95% CI)	P	N	/1000 py (95% CI)	HR (95% CI)	P
Total	1174				2387				2387			
Unadjusted												
Physical frailty	72/496	14.5	1.89 (1.30–2.73)	0.001	31/1154	2.7	2.59 (1.35–4.98)	0.004	268/1154	21.47 (19.05–24.20)	2.29 (1.86–2.81)	< 0.001
Mental frailty	36/256	14.1	1.47 (0.97–2.22)	0.068	23/657	3.5	2.95 (1.62–5.37)	< 0.001	174/657	24.60 (21.21–28.55)	2.09 (1.72–2.55)	< 0.001
Social frailty	28/172	16.3	1.75 (1.11–2.76)	0.015	23/438	5.3	5.09 (2.79–9.28)	< 0.001	117/438	24.76 (20.66–29.68)	1.89 (1.53–2.35)	< 0.001
Adjusted^1^												
Physical frailty			1.49 (1.01–2.20)	0.044			1.54 (0.75–3.17)	0.238			1.51 (1.21–1.89)	< 0.001
Mental frailty			1.12 (0.71–1.74)	0.629			1.68 (0.86–3.26)	0.129			1.46 (1.18–1.81)	< 0.001
Social frailty			1.27 (0.83–2.08)	0.331			3.43 (1.77–6.65)	< 0.001			1.32 (1.05–1.65)	0.018
Unadjusted												
Robust without MF or SF	37/499	7.4	1.00		6/884	0.7	1.00		88/884	8.64 (7.01–10.65)	1.00	
Robust with MF and/or SF	19/179	10.6	1.48 (0.83–2.65)	0.184	7/349	2.0	3.00 (1.00–8.98)	0.050	48/349	11.82 (8.91–15.68)	1.35 (0.95–1.91)	0.099
Pre-frail or Frail without SF or MF	43/318	13.5	1.95 (1.23–3.11)	0.005	4/626	0.6	0.94 (0.26–3.35)	0.925	103/626	14.82 (12.21–17.97)	1.74 (1.31–2.31)	< 0.001
Pre-frail or Frail with MF and/or SF	29/178	16.3	2.43 (1.44–4.09)	0.001	27/528	5.1	7.89 (3.23–19.23)	< 0.001	165/528	29.84 (25.62–34.76)	3.48 (2.69–4.51)	< 0.001
Adjusted^2^												
Robust without MF or SF			1.00				1.00				1.00	
Robust with MF and/or SF			1.20 (0.66–2.19)	0.555			3.91 (1.08–14.10)	0.037			1.40 (0.99–2.00)	0.061
Pre-frail or Frail without SF or MF			1.59 (0.98–2.58)	0.059			1.33 (0.33–5.41)	0.689			1.52 (1.14–2.03)	0.005
Pre-frail or Frail with MF and/or SF			1.65 (0.95–2.87)	0.078			7.17 (2.32–22.2)	0.001			2.25 (1.70–2.97)	< 0.001

^1^Model includes: gender, age, comorbidity, physical frailty, mental frailty and social frailty

^2^Model includes: gender, age and comorbidity

### Combined PF, MF, SF dimensions and disability, nursing home referral and mortality

Using physically robust participants without SF or MF as the reference group (Tables 3 and 4), the associations of physically pre-frail and frail in combination with SF and/or MF with functional disability, severe disability, nursing home referral and mortality were examined in cross-sectional and longitudinal analyses.

### Functional and severe disability

In cross-sectional analyses (Table 3), the prevalence of functional disability was lowest among the Robust without MF or SF (16.3%) and increased progressively among the PF, MF and SF combination groups to the highest prevalence (83.3%, 55/66) among those who were Frail with MF and/or SF (OR = 15.78, *p* < .001). ORs increased among the Pre-frail or Frail without MF or SF (OR = 1.34, *p* = .036), but were greater in the Pre-frail or Frail with MF and/or SF (OR = 3.10, *p* < .001). Similar trends were found for severe disability, with the Pre-frail or Frail with MF and/or SF recording the highest OR estimate (OR = 15.37, *p* < .001). In longitudinal analyses (Table 4), the incidence of functional disability increased from 7.4% among the Robust without MF or SF to 16.3% in the Pre-frail or Frail with MF and/or SF (OR = 1.65, *p* = .078).

### Nursing home referral

Nursing home referral increased progressively from the Robust without MF or SF group (0.7%) to the highest level (5.1%) in the Pre-frail or Frail with MF and/or SF group (OR = 7.17, *p* = .001), after adjusting for age, gender and medical comorbidity (Table 4).

### Mortality

Mortality rates increased progressively from 8.6 per 1000 person-years in the Robust without MF or SF group to the highest level (29.8 per 1000 person-years) in the Pre-frail or Frail with MF and/or SF group (HR = 2.25, *p* < .001), after adjusting for age, gender and comorbidity. The addition of MF, SF or both to PF increases the mortality rate.

### Receiver operating characteristic analyses

Areas under ROC (AUCs) for PSF, PMF and PMSF were satisfactory and close in magnitude for all outcome variables (Table 5). Nevertheless, the highest AUC for each outcome variable was obtained only when all three frailty dimensions were included in analyses.

**Table 5:** Receiver Operator Characteristics of PF, SF, MF and their various combinations

	Areas Under ROC				
	Cross-Sectional Analysis		Longitudinal Analysis		
	Functional Disability	Severe Disability	Functional Disability	Mortality	Nursing Home Referral
Social Frailty (SF)	0.553 (0.533–0.573)	0.677 (0.586–0.768)	0.541 (0.503–0.578)	0.564 (0.540–0.587)	0.673 (0.598–0.748)
Mental Frailty (MF)	0.592 (0.570–0.614)	0.766 (0.692–0.839)	0.536 (0.494–0.576)	0.594 (0.568–0.620)	0.621 (0.551–0.701)
Physical Frailty (PF)	0.610 (0.587–0.632)	0.728 (0.681–0.774)	0.579 (0.533–0.624)	0.608 (0.583–0.634)	0.613 (0.544–0.682)
Physical and Social Frailty (PSF)	0.641 (0.616–0.666)	0.900 (0.829–0.971)	0.600 (0.550–0.650)	0.648 (0.618–0.677)	0.685 (0.607–0.763)
Physical and Mental Frailty (PMF)	0.654 (0.628–0.679)	0.892 (0.819–0.965)	0.597 (0.547–0.647)	0.654 (0.625–0.683)	0.656 (0.577–0.735)
Physical, Mental and Social Frailty (PMSF)	0.656 (0.630–0.682)	0.901 (0.825–0.976)	0.603 (0.552–0.654)	0.661 (0.631–0.691)	0.707 (0.628–0.786)

Footnotes: 95% confidence intervals are enclosed within brackets

## Discussion

This study of the physical, mental and social dimensions of frailty within a multidimensional framework provides empirical evidence for the clinical relevance and utility for each frailty dimension individually and when used in combination as multidimensional frailty. PF, MF and SF were operationalized using existing theoretical models [10] and independently predicted various adverse health outcomes such as functional and severe disability, nursing home referral and mortality. When compared to MF and SF, PF appears to be a stronger predictor of adverse health outcomes, concurring with findings obtained using the TFI [4]. The addition of MF and SF to PF incrementally increased risk estimates of various adverse health outcomes. This is consonant with the findings in the Girona or Spain study [28], which showed that combining various frailty dimensions increases mortality HRs; HRs associated with one frailty dimension = 1.9, with two frailty dimensions = 3.9 and with three frailty dimensions = 10.4.

The three frailty dimensions are related but distinct constructs. There were considerable overlaps of the physical, mental and social functioning components belonging to each of the three frailty dimensions. The co-occurrence of MF and PF is consistent with the known association of impaired cognitive functioning and physical decline in late life [29]. While significant overlaps were present between the three frailty dimensions, it was also interesting to note that a sizable proportion of the population exhibited PF, MF or SF in isolation. The three frailty dimensions also shared many similar associations with socio-demographic, lifestyle, and health and behavioral profiles. These commonalities were unsurprising when viewed holistically within the bio-psycho-social model of common personal, social and environmental factors influencing health, disease and functioning. However, there remain associations unique to each frailty dimension. For instance, strong associations between PF and obesity, high blood pressure, metabolic syndrome, coronary heart disease and cardiac disease may be explained by shared biological factors including neuro-endocrine, hormonal and metabolic imbalances and chronic inflammation. The strong association between MF and arthritis was also unsurprising given the characteristic immune and inflammatory pathophysiology of the chronic disease and co-morbid depression. As expected, both PF and MF were strongly associated with increased physician visits and polypharmacy, while SF was not associated with health service utilization. This may reflect the unique situation of unmet needs and low demands of an under-reached group in this study population and healthcare setting.

Frailty dimensions might change over time. For example, PF transitions have been documented [30–33]. Frailty dimensions might also influence and interact with each other. For instance, SF predicted physical and cognitive decline in Japanese community-dwelling older adults [34]. Similarly, MF might hasten the progression of PF or SF as depression, poor cognitive functioning and poor self-rated health could limit one’s physical and social activities. The above should be further investigated in future studies.

PF has been very intensively studied. Its biological underpinnings with sarcopenia provide a strong basis for developing and validating screening and assessment tools. In contrast, the social and mental dimensions of frailty are rarely investigated. A preponderance of current multidimensional measurement tools are designed as global assessment tools with a total score which is predictive of health and functional outcomes. However, with the exception of the TFI, these multidimensional scales do not provide users with domain scores. There is much value in having individual frailty scores alongside a global multidimensional frailty score. As shown in the current study, PF, MF and SF individually and in combination predicted functional disability, severe disability, nursing home referral and mortality. These findings provide empirical support for the clinical relevance and use of a holistic approach towards frailty conceptualization [10]. In addition, the availability of individual domain scores allows for tailored policies and multi-domain interventions to address frailty in aging populations. Indeed, our multidimensional frailty construct was designed to match frail elderly to targeted community interventions that might be multidimensional in nature, depending on the needs of each older adult. For instance, those with PF and MF would be encouraged to attend interventions that contain exercise and cognitive stimulation while those with MF and SF would attend interventions with cognitive and social components. As the current assessments used may still be lengthy, future work should develop briefer multidimensional frailty screening and assessment tools that are equally predictive of adverse health outcomes. Such tools can be used in community-based assessments where older adults are segmented and directed to multi-domain frailty interventions tailored to their PF, MF and SF statuses.

The observed prevalence of individual and combined frailty dimensions is notable in this Asian population study. With all but 37.0% who were robust, the prevalence of any frailty dimension was 63.0%. PF was the most common frailty dimension observed (48.3%), followed by MF (27.5%) and SF (18.3%). With the exception of PF, single domain frailty was generally less prevalent as compared to multidimensional frailty with two or more frailty domains: PF alone = 26.2%; MF alone = 7.8%, SF alone = 4.7%, multidimensional frailty = 24.2%. Multidimensional frailty with all three PF, MF and SF together was present in 7.0%. Published data on the prevalence of multidimensional frailty in various populations are scarce and are based on differing operational definitions of PF, MF and SF, rendering comparison across studies difficult [4, 28]. For instance, in Girona, Spain, the prevalence of any frailty dimension was 38.8% in a study of rural community-dwelling older residents who were aged 75 and over [28]. Interestingly, MF was more prevalent than PF while SF was the least common. In another study of community-dwelling adults aged 40 to 81 in Netherlands, the prevalence of any frailty dimension was 17.1% [35]. In their study, MF and SF were more prevalent than PF, which was the least common. More studies should be conducted using standardized operational definitions of PF, MF and SF to examine possible variations in the prevalence of each frailty dimensions across different populations and their differing impacts on health, functioning and care outcomes.

Results should be interpreted with reference to the strengths and limitations of this study. The multidimensional frailty construct was derived based on strong theoretical formulations and has been empirically shown to possess face and construct validity [4, 7]. Nevertheless, its construct validity, particularly its convergent and discriminant validity, can be further assessed in future studies. While the multidimensional frailty construct shares the same domains as the TFI, the variables used to index each domain differ across the two scales. In addition, the operationalization of multidimensional frailty could have been limited by the availability of measures. PF is widely assessed using the standard criteria proposed by Fried et al., but may use various operational measures [12]. To assess weakness and slowness, we used proxy measures derived from the Performance Oriented Mobility Assessment of gait balance and control that was available in SLAS-1. We have shown in an independent study using knee extension strength and gait speed from six-meter walk in the second wave SLAS cohort (SLAS-2) that the two different operationalization of PF have good agreement (weighted kappa = 0.63) and were equally and strongly predictive of adverse health outcomes. The operationalization of SF was in line with a recent scoping review, which defined SF as a continuum of being at risk of losing, or having lost general or social resources, social behaviors and activities and self-management abilities that are important for fulfilling one’s basic social needs [14]. This moves beyond narrowly defining SF as the lack of social participation and perceived lack of social contacts and support. For MF, there is general consensus that this term may be applied to the declining mental abilities of older adults, involving cognition, mood and motivation as a consequence of loss of brain functional reserve under stress [13, 36]. PF, MF and SF measures could be standardized along these criteria in future studies to allow for comparison across different populations worldwide.

Some measurement artifacts may exaggerate observed overlaps across frailty dimensions, such as that between PF (due to exhaustion) and MF (due to depression). However, they did not appear to lessen the distinctions among the three frailty dimensions. It should be pointed out that frailty dimension measures in this study were elaborate and not designed to be brief screening tools, such as the TFI or EFS. There is thus room to develop brief screening and assessment tools for multidimensional frailty. Future studies should also investigate transition of MF and SF statuses, as has been shown for PF [30–33].

Singapore’s nursing home context and the nature of collected data should be considered when interpreting results for nursing home referral. Family and social service factors (limited access to affordable nursing home services despite lack of family caregiver) play a substantial role in prioritizing nursing home referral and placement in Singapore. PF and MF were not significant predictors of nursing home referral. This was partly explained by low statistical power from the unsurprisingly low incidence of nursing home referral in the study sample. There was also the possible effect of the time lag between baseline frailty measurements (2003–2005) and nursing home referral data (2010–2018). PF could have changed during this time lag, given its dynamic nature [30–33]. Likewise for MF, due to transitions in participants’ cognitive function, mood and self-rated health. Changes in SF could be less as it is partially made up of stable social resource factors. The low incidence of nursing home referral, low statistical power and time lag effect might thus account for the lack of significant associations between PF and MF and nursing home referral. Nevertheless, SF significantly predicted increased nursing home referral, strongly attesting to its powerful prediction of long-term institutional care.

AUCs obtained in ROC analyses were satisfactory but not large. Figures obtained were within the expected range given that the predicted outcome variables (e.g., mortality, disability) are influenced by a number of factors other than frailty. This is not unique to the current study. For reference, similar AUCs were obtained when the Framingham risk score, designed to predict development of cardiovascular disease, was used to predict the risk of chronic kidney disease in the absence of renal parameters [37].

## Conclusion

Compared to PF alone, a multidimensional bio-psycho-social approach to frailty increases its ability to predict adverse health outcomes, such as disability, nursing home referral and morality, amongst Chinese Singaporean community-dwelling older adults. This study highlights the clinical relevance and utility of multidimensional frailty screening tools in the design of targeted multi-domain community-based health and social interventions to reduce frailty in community-dwelling older adults. These results also emphasize the need for upstream preventive health and social services to contain disability burden.

## Data Availability

Data is not available for online access, however readers who wish to gain access to the data can write to the senior author Prof Ng TP at: “pcmngtp@nus.edu.sg”. Access can be granted on reasonable request and subject to the Institutional Review Board and the research collaborative agreement guidelines for the study. This is a requirement mandated for this research study by our IRB and funders.

## Abbreviations

ADL: Activities of Daily Living
AUC: Area under ROC
CMMSE: Chinese version of the Mini-Mental State Examination
EFS: Edmonton Frail Scale
HR: Hazard Ratio
IADL: Instrumental Activities of Daily Living
MF: Mental Frailty
MOH: Ministry of Health
OR: Odds Ratio
PF: Physical Frailty
PMF: Combined PF-MF Dimensions
PMSF: Combined PF-MF-SF Dimensions
PSF: Combined PF-SF Dimensions
ROC: Receiver Operating Characteristic
SF: Social Frailty
SLAS-1: First cohort of the Singapore Longitudinal Aging Studies
SLAS-2: Second cohort of the Singapore Longitudinal Aging Studies
TFI: Tilburg Frailty Indicator
